# Computational modeling reveals a key role for polarized myeloid cells in controlling osteoclast activity during bone injury repair

**DOI:** 10.1038/s41598-021-84888-1

**Published:** 2021-03-15

**Authors:** Chen Hao Lo, Etienne Baratchart, David Basanta, Conor C. Lynch

**Affiliations:** 1 Tumor Biology Department, SRB3, Moffitt Cancer Center and Research Institute, 12902 Magnolia Drive, Tampa, FL 33612 USA; 2 Integrated Mathematical Oncology Department, SRB4, Moffitt Cancer Center and Research Institute, 12902 Magnolia Drive, Tampa, FL 33612 USA; 3 Oncology and Cancer Biology PhD Program, University of South Florida, Tampa, FL USA

**Keywords:** Computational models, Data integration, Population dynamics, Innate immune cells, Osteoimmunology, Bone, Computer modelling, Differential equations, Dynamical systems, Multicellular systems, Population dynamics

## Abstract

Bone-forming osteoblasts and -resorbing osteoclasts control bone injury repair, and myeloid-derived cells such as monocytes and macrophages are known to influence their behavior. However, precisely how these multiple cell types coordinate and regulate each other over time within the bone marrow to restore bone is difficult to dissect using biological approaches. Conversely, mathematical modeling lends itself well to this challenge. Therefore, we generated an ordinary differential equation (ODE) model powered by experimental data (osteoblast, osteoclast, bone volume, pro- and anti-inflammatory myeloid cells) obtained from intra-tibially injured mice. Initial ODE results using only osteoblast/osteoclast populations demonstrated that bone homeostasis could not be recovered after injury, but this issue was resolved upon integration of pro- and anti-inflammatory myeloid population dynamics. Surprisingly, the ODE revealed temporal disconnects between the peak of total bone mineralization/resorption, and osteoblast/osteoclast numbers. Specifically, the model indicated that osteoclast activity must vary greatly (> 17-fold) to return the bone volume to baseline after injury and suggest that osteoblast/osteoclast number alone is insufficient to predict bone the trajectory of bone repair. Importantly, the values of osteoclast activity fall within those published previously. These data underscore the value of mathematical modeling approaches to understand and reveal new insights into complex biological processes.

## Introduction

Bone healing subsequent to injury or trauma is a significant clinical problem in orthopedics and rehabilitation^1–3^. Understanding the processes involved and how cells coordinate and control each phase of bone injury repair can reveal opportunities to accelerate healing and improve patient outcomes while reducing cost. The phases of bone repair in diaphyseal, epiphyseal or metaphyseal fractures have been well characterized^1,4–7^. For example, in critical non-union fractures such as internal injuries to the supporting trabecular bone architecture, a rapid inflammatory response is followed by mass callus formation spanning the periosteal and endosteal surfaces. The callus is then mineralized by infiltrating mesenchymal stromal cells (MSCs) that differentiate into cartilage, and bone-forming chondrocytes and osteoblasts respectively^1,2^. Subsequently, activated osteoclasts mediate resorption and clearing of the ossified callus reestablishing the marrow trabeculae^1^. In addition to osteoblasts and osteoclasts, other cell types are also involved in the bone healing/remodeling process, such as resident and infiltrating immune cells that exert pro- and anti-inflammatory activities depending on environmental cues^1,8–10^. This is evidenced by the fact that acute pro-inflammatory factor administration (e.g. TNFα) can improve bone repair while prolonged administration has the opposite effect^11–15^. Monocytes and macrophages are major components of the bone immune infiltrate subsequent to injury^1,8,9,16^. Previous studies using genetic or pharmacological depletion of myeloid cells such as macrophages demonstrated significantly delayed time to bone repair ^10,16–19^. However, precisely how these multiple cell types coordinate and regulate osteoblast and osteoclast activity over time is challenging to dissect using traditional in vitro and in vivo biological approaches.

A potential approach to overcome this hurdle is the integration of experimental data with computational models that allow for the analysis of multiple cell types at any time point during bone injury repair. Previous reports, including from our group, have successfully demonstrated the feasibility of mathematical modeling approaches to enhance our understanding of how cells interact in the bone ecosystem to coordinate homeostasis and cancer-bone interactions^20–29^. There are a number of mathematical model approaches that can be employed such as ODEs that can be used to model bone cell populations in normal and disease processes^30–35^. Individual cellular dynamics can also be considered by representing the cell populations as either a continuous spatial field whose dynamics are described by a set of partial differential equations (PDE)^36,37^, or as individual agents in an agent-based model approach^30^. Although these models have been used to examine bone injury repair and homeostasis, they have largely focused on the interaction between bone-building osteoblasts and bone-resorbing osteoclasts^30–32,34,37^. Some models have considered immune populations but these are theoretical and are not driven by biological data that provides quantitative information for each population and various timepoints throughout the bone injury repair process^38,39^.

To address this, we used an in vivo model of bone injury to longitudinally measure changes in pro- and anti-inflammatory monocytes and macrophages in addition to osteoblast and osteoclast numbers and bone volume around the site of injury. We then used the obtained biological data combined with empirically-derived parameters from the literature to power an ODE model of trabecular bone injury repair and examine the impact of infiltrating immune cells on osteoblast and osteoclast activity over time in regard to trabecular bone volume dynamics. The ODE model generated herein, demonstrated that the temporal interplay between myeloid-derived pro- and anti-inflammatory populations are critical in driving osteoblast and osteoclast response but interestingly, using a constant rates of bone formation and resorption, the mathematical model failed to recapitulate the trabecular bone volume dynamics. Further interrogation of the model demonstrated that the rate of osteoclast resorptive activity must vary greatly over the course of injury resolution to return the bone volume to homeostasis. This insight has not been considered to date and underscores the value of mathematically modeling complex multicellular biological process.

## Results

### Osteoclast and osteoblast numbers fluctuate dynamically in response to bone injury

**Figure 1:**
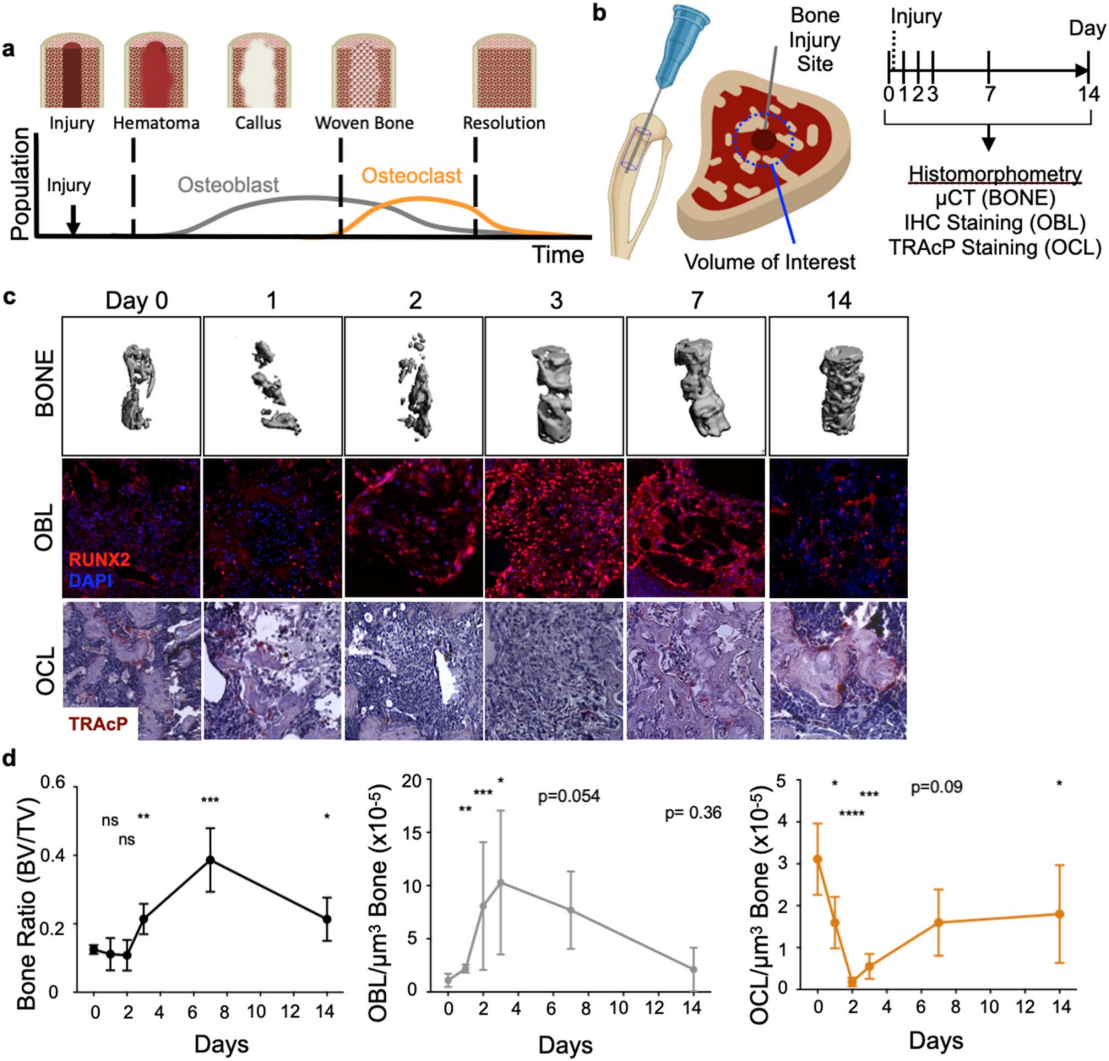
Osteoblast (OBL) and osteoclast (OCL) numbers temporally fluctuate dynamically as bone heals from injury. (**a**) Schematic summarizing published dynamics of OBL and OCL following bone injury. (**b**) Schematic depicting the experimental workflow to induce bone injury in mice and generate bone, osteoblast and osteoclast dynamic data. (**c**) Representative images of micro-computed tomography revealed trabecular bone status (BONE). Decalcified bones were stained and quantified for OBL by RUNX2 immunofluorescence staining (OBL), and OCL by tartrate-resistant acid phosphatase (TRAcP) staining (OCL). (**d**) Quantitation of temporal dynamics of bone volume, osteoclast and osteoblast population.

The stages and duration of trabecular repair following non-critical/ non-displaced bone fractures largely follow the same program, whereby subsequent to injury, early inflammation and hematoma occur rapidly, followed by the formation of a callus that is subsequently mineralized by bone-forming osteoablasts^4,5,40^. The callus is then remodeled via the activity of bone-resorbing osteoclasts^1,18,41–46^ (Fig. 1a). Osteoblasts and osteoclasts are critical mediators of these steps and their numbers shift accordingly during each phase of repair. Existing theoretical models of bone remodeling assume osteoblast and osteoclast activities are constant over time and their numbers, therefore, directly predict bone dynamics^31,32,34,37^. To evaluate this prevailing assumption, we first asked if modeling osteoclast and osteoblasts alone was sufficient to accurately predict corresponding bone remodeling dynamics using experimental data. We focused on modeling trabecular bone dynamics as opposed to the cortical surfaces given that these injuries resolve quickly over a shorter time period compared those involving cortical bone. To generate parameters to power such an ODE model, we used an experimental model of trabecular bone injury repair: non-critical trabecular disruption resulting from direct intratibial penetration via the knee epiphysis into the medullary canal^47–50^ (Fig. 1b). Tibias from mice were collected prior to injury at baseline (day 0), and at day 1, 2, 3, 7 and 14 (n = 5 mice/time point) following injury. High-resolution micro-computed tomography (μCT) analysis of uninjured tibia established baseline bone volume (BV/TV) (Fig. 1c and d). Our data show that after injury, trabecular bone volume around the injury site diminished over a 48-h period, prior to a robust increase in mineralized bone content between days 2 and 7. By day 14, the bone volume returned toward baseline values. We directed our μCT and histological analyses on the area surrounding the bone injury rather than the entire bone since our goal was to quantify cellular dynamics and changes in the bone marrow specifically in response to trabecular injury; values that could be diluted by measurements in non-injured areas of the medullary canal as well as the cortex (Supplemental Fig. 1a and b). Focusing on the site of injury and surrounding area (in keeping with parameters from the μCT analysis), histologically, we observed sequential increases in osteoblasts followed by osteoclasts, findings that are qualitatively compatible with our BV:TV μCT analyses and are in line with previous published observations that have also quantitated trabecular dynamics^50–52^ (Fig. 1c and d).

### Bone repair dynamics cannot be computationally recapitulated using constant osteoblast and osteoclast activity rates

To date, bone resorption and formation rates have been difficult to measure in vivo. Despite various in vitro studies showing that osteoblast and osteoclast activity can be controlled by inflammatory factors and cytokines^15,53–64^, existing theoretical mathematical models of bone remodeling largely assume that resorption and formation rates per cell are fixed/constant over time. Since measuring whether osteoblast and osteoclast activities vary over time in vivo during bone injury repair is experimentally challenging, we employed an integrated experimental and mathematical approach to address this knowledge gap.

**Figure 2:**
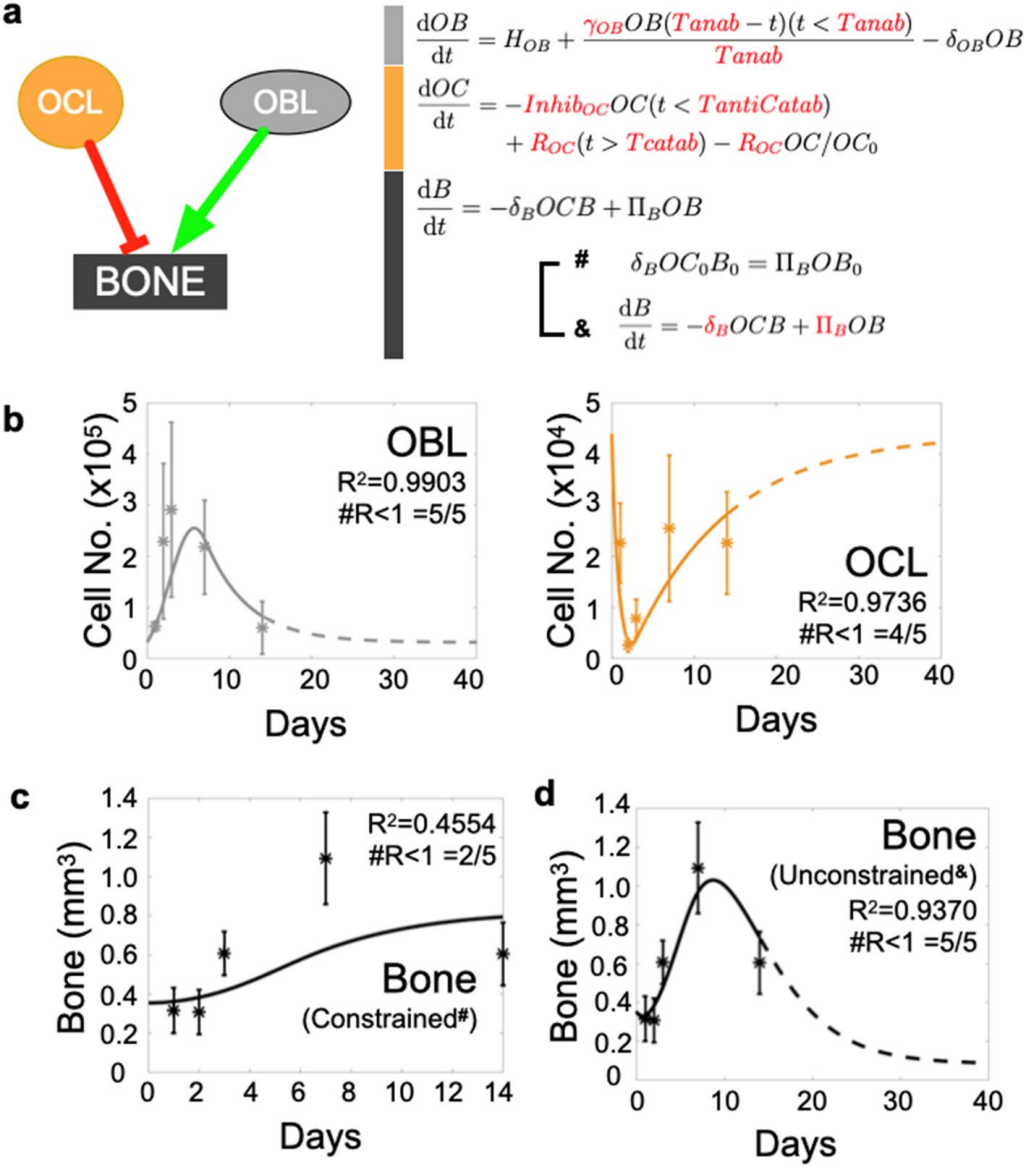
Osteoblast (OBL) and osteoclast (OCL) activities as measured at homeostasis do not allow accurate bone prediction during injury repair in vivo. Histological quantitation of tibia bones parameterizes mathematical ordinary differential equation (ODE) model of bone injury repair. (**a**) Ordinary differential equations (ODE) describing dynamics of OCL and OBL population are paired with published parameters to form an initial ODE model to predict bone repair dynamic. Schematic depicts OCL resorb (red line) and OBL form bone (green line). ODE expressions with unknown value (red) were estimated as the model optimizes fits to in vivo data. (**b**) Model produces accurate fits to OCL and OBL dynamics. (**c**) Model falsely predicts bone dynamics given OBL and OCL fits in the first 14 days following bone injury when it samples various publication-derived OCL resorption rates (each dashed line represents one sampling). OBL bone formation rates are mathematically estimated in each sampling to ensure predictions will eventually return to homeostasis (**#**). (**d**) Alternatively, ODE model was allowed to freely seek out a combination of resorption and formation rates to best fit data within the 14-day time period (**&**).

Using the obtained biological data and publicly-available parameter values regarding osteoblast and osteoclast behavior (Fig. 1c, Supplemental Fig. 2 and Table 1), we developed an initial data-driven mathematical ODE model to recapitulate the control of trabecular bone restoration exclusively by these two populations (Fig. 2a). This initial ODE model simulated the bone injury event as a transient osteoblast (OBL) expansion and a decrease in osteoclast (OCL) population from day 0 to 2 (see mathematical and computational methods). Fits to the rest of the OCL and OBL population data were optimized within the parameter space defined by published literature, such as regarding cellular lifespan and proliferation rates (Fig. 2b and Table 1). Optimal fits with greatest R^2^ value and number of residuals less than 1 (#R < 1) were subsequently used for estimating bone volume dynamics. Using these OCL and OBL optimized fits, the ODE model attempted to recapitulate experimental bone dynamics by sampling *constant* bone resorption rates within a range previously described in literature^50,53,65,66^. A corresponding bone formation rate was estimated in each sampling as to ensure a return to baseline bone volume at the end of the injury repair process (Fig. 2a **#**). Interestingly, using this iterative approach, the ODE predictions largely overestimated the bone volume dynamics compared to the experimental data (Supplemental Fig. 3). In fact, the best-fitted iteration, which used the lowest published OCL resorption rates^66^, only achieved an R^2^ value of 0.4554, and #R < 1 of 2/5 (Fig. 2c). This indicated that either published measurements of in vitro bone resorption/formation parameters do not reflect in vivo rates, and/or that bone resorption/formation rates by osteoclasts and osteoblasts are variable over time during the course of injury repair. To address this, we alternatively fitted the model to bone dynamics data while allowing the optimization algorithm to freely determine an optimal combination of *constant* bone resorption and formation rates that were not forced to return to baseline bone volume subsequent to injury (Fig. 2a **&**). This resulted in improved bone volume dynamic fits during injury repair but, of note, the final bone volume reached by the ODE was 70% lower compared to that of baseline (Fig. 2d). Taken together, these data suggest that osteoclast and osteoblast activity rates must vary greatly during injury response in order to return the trabecular bone to homeostasis during the injury repair time-frame. This raised the question as to what cellular/environmental cues are potentially responsible for controlling their activity.

**Table 1:** Parameters extracted from published literature were combined with temporal dynamics data in ODE model to estimate previously unknown parameters needed to fit bone data.

Parameter	Description	Value	Unit	Supplemental references	SE
*δ*_*OB*_	Osteoblast lifespan	0.19	Day^−1^	*33, 34*	–
*γ*_*OB*_	Osteoblast proliferation rate	0.873	Day^−1^	Estimated	0.1999
*Inhib*_*OC*_	Osteoclast inhibition	1.2186	Day^−1^	Estimated	0.3540
*R*_*OC*_	Osteoclast recruitment	6774.8	Cell Day^−1^	Estimated	1967.6
*T*_*anab*_	Duration of anabolism	6.6924	Day	Estimated	1.8649
*T*_*antiCatab*_	Osteoclast Inhibition duration	2	Day	Estimated	–
*T*_*Catab*_	Starting time of catabolism	2	Day	Imposed	–
	Published resorption rate range^#^	[1 × 10^**−8**^ − 5 × 10^**−5**^]	mm^3^ Cell^−1^ Day^−1^	26, 27, Imposed	–
*δ*_*B*_	Per bone volume unit resorption rate^#^	7.034 × 10^−6^	Cell^−1^ Day^−1^	Estimated	7.30 × 10^***−***7^
*Π*_*B*_	Bone formation rate^#^	–	mm^3^ Cell^−1^ Day^−1^	Determined from *δ*_b_	–
*δ*_*B*_	Per bone volume unit resorption rate^&^	5.7687 × 10^−6^	Cell^−1^ Day^−1^	Estimated	1.63 × 10^**−6**^
*Π*_*B*_	Bone formation rate^&^	1.2164 × 10^−6^	mm^3^ Cell^−1^ Day^−1^	Estimated	1.6043 × 10^−7^

Apposition rates from confined model (#) were calculated in fashion to offset resorption rates derived from publication, to maintain constant bone volume at homeostasis.

### Polarized pro- and anti-inflammatory monocytes and macrophages emerge in distinct temporal waves during bone injury repair

**Figure 3:**
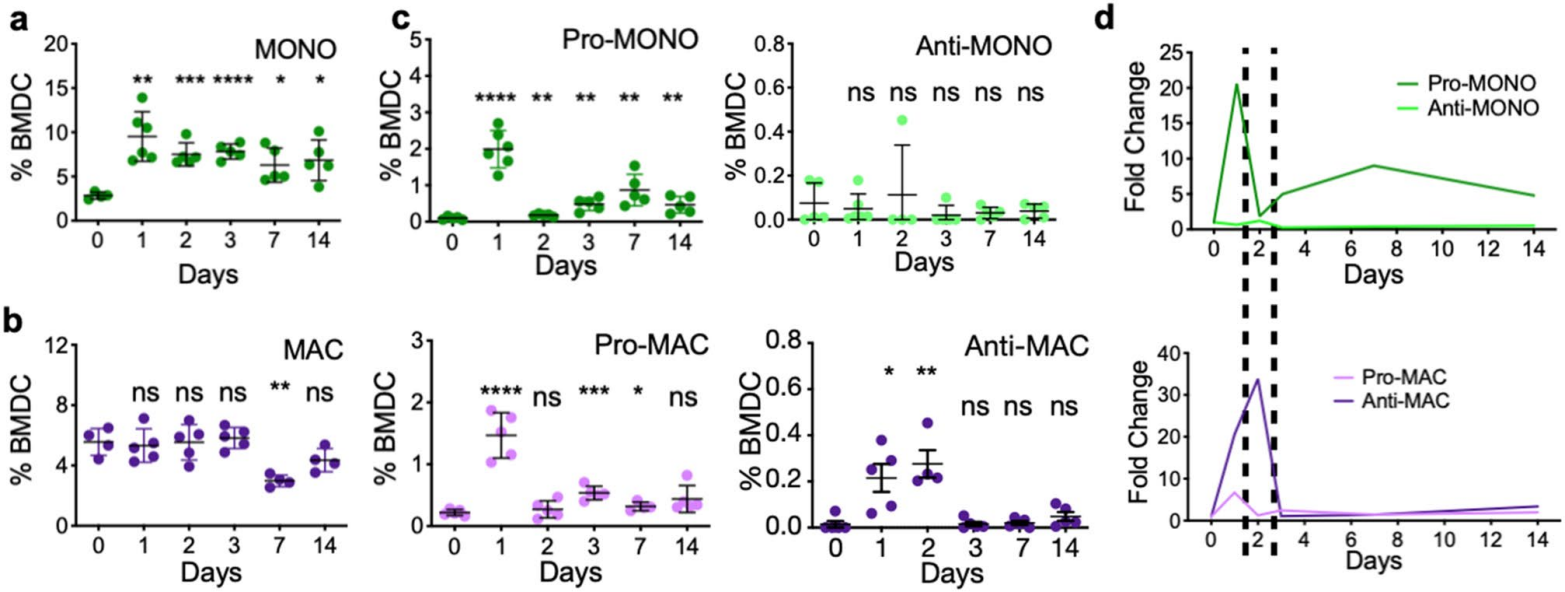
Transient waves of pro- and anti-inflammatory monocytes and macrophages alternate dynamically during bone injury repair. Flow cytometry performed on tibia bone marrow harvested from C57BL/6 mice at various time points after injury (n = 30; 5/time point) reveals diverse myeloid dynamics and polarization. Time points corresponds to time points from histological data. Total monocyte (CD11b + LY-6C^HI^ LY-6G-; (**a**) and macrophage (CD11b + LY-6C^LO^ LY-6G-; (**b**) and their respective pro- and anti-inflammatory subsets (**c**) each uniquely fluctuates following bone injury (Student t-test compares all time points to its Day 0 for each subset; *p < 0.05 **p < 0.005 ***p < 0.0005 ****p < 0.00005 ^ns^*p* > 0.05). (**d**) Temporal dynamics of pro and anti-inflammatory monocytes and macrophage numbers are normalized as fold change relative to levels at homeostasis. Dashed lines show timings of pro- and anti-inflammatory polarization are mutually exclusive.

Monocytes and macrophages are key cellular species in the bone ecosystem and their pro- and anti-inflammatory functions have been implicated in the bone injury repair process and in the regulation of osteoblast/osteoclast activity^8,18,19,50,67,68^. Studies have shown, for example that, (1) myeloid cells are polarized in bone injury and inflammation, (2) pro-inflammatory factors and myeloid cells stimulate osteoclast activity, and (3) anti-inflammatory/wound-healing factors and myeloid cells stimulate osteoblast activity (Supplemental Fig. 4)^1,9,11,45,51,67–69^. Based on this rationale, we therefore hypothesized that fluctuations in the number and polarization status of myeloid populations control osteoclast and osteoblast activity during bone repair. To test this hypothesis, we reanalyzed the non-critical trabecular bone injury experiment. Tibias from mice were collected at baseline prior to injury (day 0), and at day 1, 2, 3, 7 and 14 (n = 5/time point) post-injury. Flow cytometry was used to measure changes in myeloid populations over time since it allowed for multiplexed analysis of phenotypic as well as polarization markers for resolving various myeloid subsets^8,17,70–80^ (Fig. 3a–c and Supplemental Fig. 5). Our results show that there are significant increases in pro-inflammatory monocytes and macrophages within the first 48 h that are subsequently rapidly depleted upon the infiltration of anti-inflammatory macrophages between 24 and 72 h (Fig. 3c). Our follow-up histological analyses using tissue sections from days 2 and 3 confirm the presence of pro- and anti-inflammatory cells at the injury site, which were not observed at baseline (Supplemental Fig. 6a and b). Interestingly, in accordance with observations from other in vivo studies, we observed a smaller second wave of pro-inflammatory monocytes between days 6 and 8^81–83^ (Fig. 3c and d).

### Integration of polarized myeloid cells control of bone remodeling activity recapitulates bone healing dynamics

**Figure 4:**
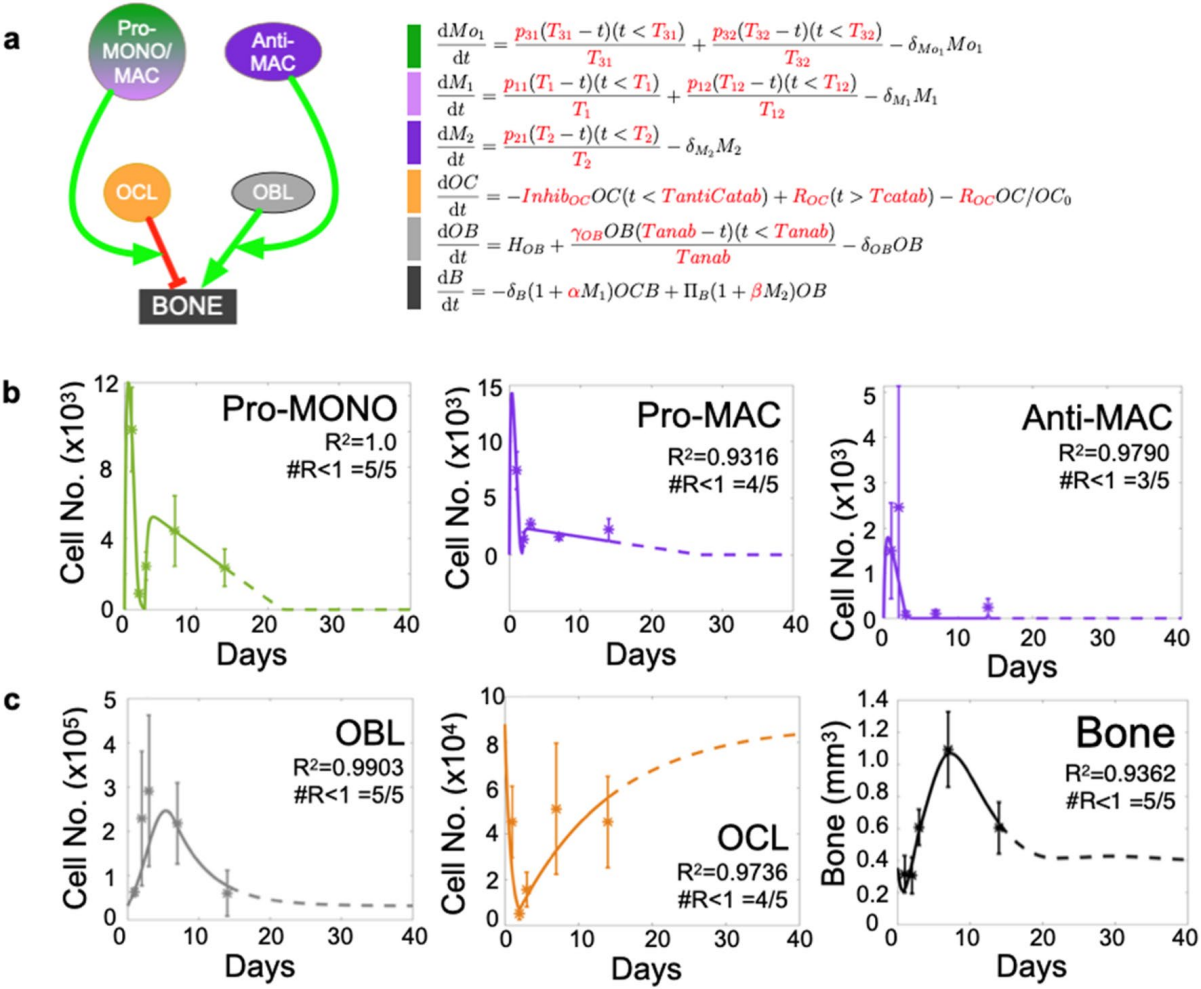
Integration of pro- and anti-inflammatory myeloid populations to modulate OCL and OBL activity sufficiently improves model fit to experimental bone data. (**a**) ODE expands to six populations and allows manually-fitted pro- and anti-inflammatory cells to enhance bone resorption and formation rates, respectively. Individual equations are shown next to schematic of ODE framework, model estimates amount of influence polarized myeloid cells have on bone remodeling activity to optimize fit to data (expressions in red). (**b**) Manual fits to pro- and anti-inflammatory monocytes (Pro- and Anti-MONO, respectively), anti-inflammatory macrophages (Anti-MAC) are represented by solid lines through error bar of data. (**c**) Myeloid data was used to predict bone dynamics given OBL and OCL fits. Statistical analysis of resulting fits on OCL, OBL and bone are shown (R^2^).

Previous studies have reported pro- and anti-inflammatory myeloid control of osteoclast and osteoblast activity; however, these observations are largely derived from in vitro settings^54,67,84–93^. To address this, we integrated the experimental quantitative data collected from each of these populations via flow cytometry into the framework of the ODE model (Fig. 4a and b). Specifically, we allowed osteoclast activity to be stimulated from baseline in proportion to the presence of pro-inflammatory cells by a model-estimated constant factor of α. Likewise, we allowed osteoblast activity to be stimulated from baseline in proportion to the presence of anti-inflammatory cells by a model-estimated constant factor of β. These assumptions are based on empirical data from published in vitro experimental data^54,67,84–86,88–93^. We then asked the expanded ODE model to optimize for levels of α and β that are needed to recapitulate bone volume dynamics. Of note, we did not integrate anti-inflammatory monocyte data as the experimental data demonstrated this population remains consistently low levels that did not fluctuate throughout the course of bone injury repair (Fig. 3c). Importantly, in our model optimization, the range of osteoblast and osteoclast activities that could be influenced by infiltrating myeloid cells were limited to published values (Supplemental Fig. 2). Given these restraints, the model nevertheless estimated an optimal set of parameters that significantly recapitulated the bone volume dynamics (R^2^ = 0.9362; #R < 1 = 5/5) (Fig. 4c). The optimized model reveals that while osteoblast activity remains relatively constant, osteoclast activity changes dramatically over time. Furthermore, in this expanded ODE model, the bone volume returned to baseline levels subsequent to injury, underscoring the biological validity of our model assumptions and reinforcing myeloid-derived infiltrating cells as important regulators of osteoblast, and in particular, osteoclast activities in the process.

### Osteoclast resorption activity does not correlate with osteoclast number during bone injury repair

**Figure 5:**
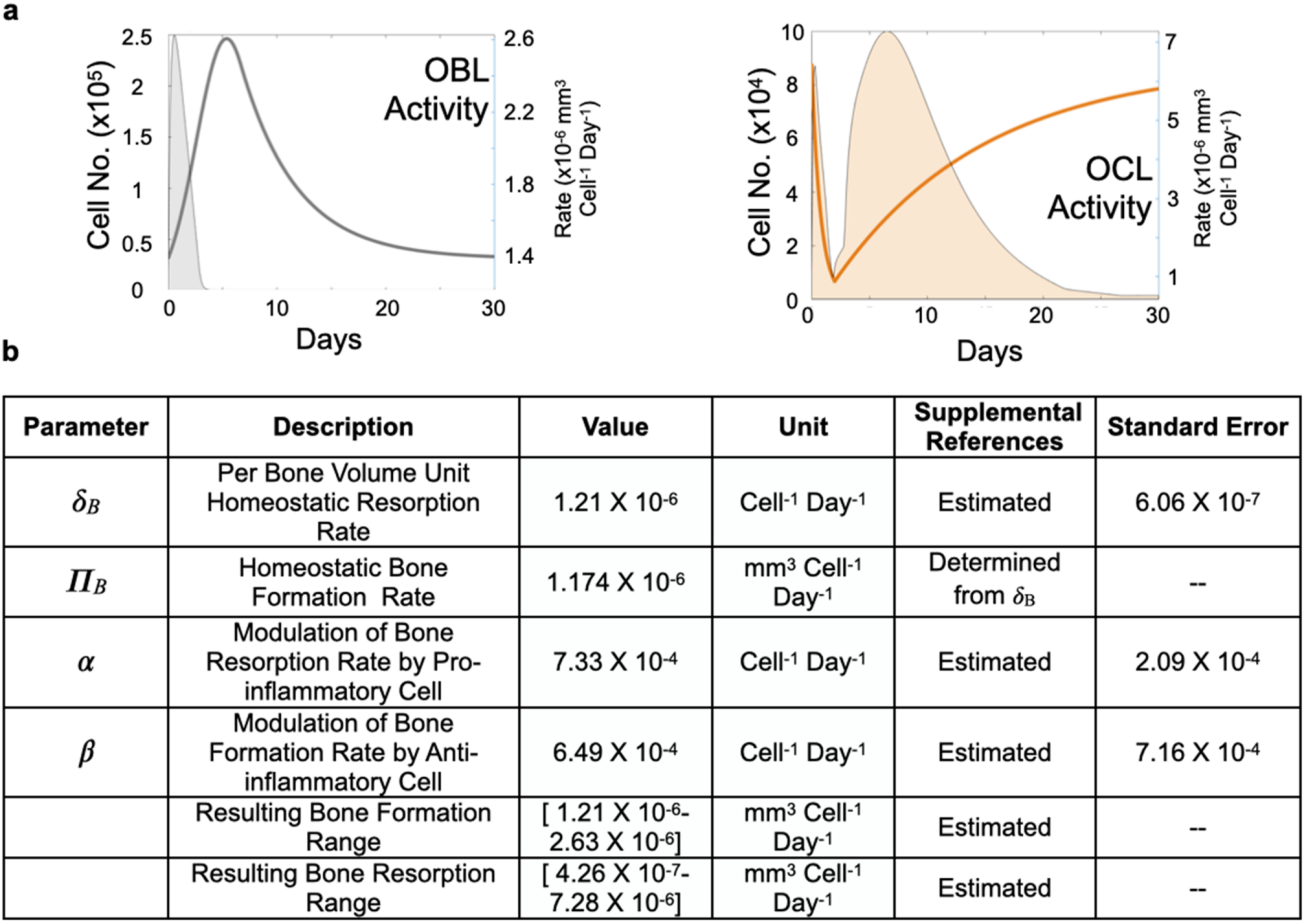
OCL and OBL activities and numbers do not correlate and vary distinctly across in bone injury repair. (**a**) OBL and OCL activity rate dynamics (filled curves plotted on the right y-axis) are plotted against their population dynamics (unfilled curves plotted on the left y-axis). Activity is temporally distinct from population dynamics, both combine to recapitulate bone dynamics. (**b**) Table detailing the known parameters used by model to estimate/infer unknown parameters.

Upon further analysis of the results generated by our expanded ODE model, we noted a disconnect between the dynamics of osteoblast and osteoclast activity versus their population numbers (Fig. 5a). The model predicts that osteoblast mineralizing activity varies slightly over time 1.21 × 10^−6^ to 2.63 × 10^−6^ mm^3^/cell/day; however, the model predicts that a range of 4.26 × 10^−7^ to 7.28 × 10^−6^ mm^3^/cell/day is required for osteoclast activity (Fig. 5a and b). These data suggest that, while osteoblast activity only increases by twofolds, a 17-fold increase in osteoclast activity is required to recapitulate injury dynamics and also return to the bone volume to homeostasis. Importantly, the noted ranges for osteoclast activity fall within those values reported in independent studies^53,65,66^ (Fig. 5b and Supplemental Fig. 2). Our model is the first to posit that the rate at which osteoclast resorbs mineralized matrix in bone healing can vary greatly depending on cues from the surrounding microenvironment.

We also submit that this variation in resorptive activity can be recapitulated by the integration of infiltrating pro- and anti-inflammatory monocytes and macrophages. To this end, we returned to our initial ODE model (Fig. 2) and asked how would osteoclast activity change overtime in order to fit to the bone data. In this agnostic approach, we no longer restricted osteoclast resorption to constant rates over time, but defined a piecewise linear function of time for osteoclast resorption rate (Supplemental Fig. 7a and b, Mathematical and Computational Methods). Independent of the parameters chosen for the initial piecewise slope conditions, the optimization algorithm identified a functional form composed of two waves: initially intense and transient between days 1 and 2, followed by a milder but persistent wave starting after Day 3 (Supplemental Fig. 7c; orange line). We noted this temporal profile was very similar to that of experimental data regarding the pro-inflammatory monocytes and macrophages populations (Fig. 3c and Supplemental Fig. 7c). While this result does not discount the possible contribution from other cells within the bone marrow, it does further supports that pro-inflammatory cells contribute significantly to osteoclast behavior and therefore bone healing dynamics (Supplemental Fig. 7). We used a similar approach to determine variable osteoblast activity and defined a piecewise linear function of time, for the bone formation rate (Supplemental Fig. 8a). Interestingly, this model did not recapitulate bone volume dynamics despite the freedom to change bone formation rate over time (Supplemental Fig. 8b; R^2^ = 0.6796). These data further support a role for pro-inflammatory myeloid cells in controlling osteoclast, and less so osteoblast activity, and therefore bone volume during injury repair.

## Discussion

The complex cellular mechanisms that control bone injury repair can be difficult to dissect given the complexity of the bone marrow microenvironment using traditional biological approaches but key insights have been made. For example, genetic and pharmacologic approaches reveal that macrophages play important roles in bone healing as well as osteoclast differentiation^51,68,93–95^. Yet, how macrophage populations quantitatively interact with each other or other cell types in the bone environment directly or indirectly over time can be challenging to identify with this approach. For instance, though polarized macrophages have been observed at sites of bone injury alongside osteoblasts and osteoclasts (Supplemental Fig. 6b)^8,11,17^, the rates at which polarized macrophages stimulate the activities of these bone cells remained difficult to evaluate and furthermore, quantitate. Computational approaches allow simultaneous interrogation of multicellular systems in which a mathematical model can infer parameter values that may be otherwise unknown. Despite this advantage, existing mathematical models of bone remodeling largely focus only on osteoclast, osteoblast and the bone; and those which integrate additional populations are theoretical^28,30–38^. As shown here, we have integrated both our experimental and published data into a mathematical framework which models interactions between myeloid cells, osteoclasts and osteoblasts, and the bone from published literature. Our findings are derived solely from the experimental in vivo bone injury model we have described herein. We are thus collaborating with orthopedic experts in examining the relevance/application of our findings in more established models of bone fracture and are considering integrating new biological parameters into our mathematical models. Nonetheless, using this model, we have concluded that bone repair cannot be recapitulated if we assume osteoclast osteoblast activities are constant over time. Our initial ODE model failed to derive accurate bone fits despite using both published and freely estimating *constant* activity rates. Therefore, based on the literature, we subsequently focused on myeloid-derived monocytes and macrophages that have noted roles in contributing to bone injury repair^51,68,93–95^, and the expanded ODE demonstrated that the dynamic waves of polarized monocytes and macrophages and their temporal control of bone remodeling activity sufficiently allowed for the accurate recapitulation of bone repair.

Another major finding from our analyses is the extent to which osteoclast resorptive activity can be modulated subsequent to osteoblast mineralization of the injury site. Existing empirical data have recorded osteoclast resorptive activities in the range of 1 × 10^−8^ to 5 × 10^−5^ mm^3^/cell/day^53,65,66^. Here, our estimations suggest that osteoclast activity varies by 17-fold magnitude over time within this published range and that pro-inflammatory monocyte and macrophages are critical for regulating this effect. Beyond our experimental model, in other critical bone injury contexts, it is possible that bone remodeling activity may vary even more. These data underscore how mathematical modeling can provide important biological insights. It should be noted that though other mathematical models have been proposed to explore mechanisms of bone repair dynamics^30,32,34,36,38^, the study presented herein, to our knowledge, is the first to leverage longitudinal biological data on multiple cellular populations and integrate this information into a mathematical model.

Additional quantitative insights provided by the ODE model include estimations on monocyte and macrophage proliferation rates as well as the rates at which pro- and anti-inflammatory cells polarize and modulate osteoclast and osteoblast activity, respectively, during the repair process. This information can be critical for therapies that target specific myeloid populations during bone injury repair in a bid to accelerate bone healing. Our study also reveals rapid expansion of pro-inflammatory monocytes and macrophages in the first 24 h with anti-inflammatory macrophages emerging shortly thereafter and persisting for up to 48 h. Interestingly, pro-inflammatory cells moderately rebound upon the clearance of anti-inflammatory cells (between days 6 and 8, Fig. 3c), suggesting a second wave of inflammation that is in keeping with other reports^81–83^. Conflicting reports suggest this could be due to (1) emergence of anti-inflammatory macrophages having an inhibitory effect on pro-inflammatory population, or (2) myeloid plasticity and repolarization^68,72,96–101^. Our next efforts with the ODE generated herein will focus on the interplay between macrophages and how their polarization states control not only each other, but also how osteoblasts and osteoclasts coordinate bone injury repair.

One caveat of our study is that the flow cytometric analysis is performed on cells isolated from the whole bone marrow, as opposed to only the volume of interest in histological datasets (See Supplemental Fig. 1). An alternative could be to perform multiplex image cytometry of the site of injury for the various myeloid populations of interest. We suspect that, while this would allow for more accurate quantitation of the myeloid cell populations infiltrating the site of information, the overall trends and shifts in those populations over time would remain similar to our flow cytometry data. To this point, our initial immunofluorescence analyses demonstrated the presence of pro- and anti-inflammatory cells during injury repair and indicate a spatial temporal element to myeloid dynamics which our ODE approach does not address (Supplementary Fig. 6). This information, in addition to other important bone parameters such as trabecular architecture (Supplementary Fig. 1b) could be used in the future for the development of hybrid cellular automaton models to investigate the spatial relationship between each population being studied. Further, our model also does not consider the potential roles of other cell types in the bone ecology that could contribute, such as T cells. Our results suggest that modeling myeloid populations provides enough resolution to satisfactorily explain the process of non-critical trabecular bone injury repair. However, other cell populations which (1) exhibits the same temporal dynamics as pro-inflammatory myeloid cells (Fig. 3C) or from the piecewise linear function analysis (Supplemental Fig. 7C) and (2) can stimulate osteoclast activity may also contribute to bone healing dynamics. Importantly, our unbasied functional form search (Supplemental Figs. 7 and 8) yielded osteoclast activity dynamics that qualitatively resonated with the population dynamics of pro-inflammatory monocytes and macrophages, supporting the importance of the myeloid population in regulating bone volume resorption. Our theoretical framework is flexible enough however that the effects of other immune cells such as T cells could be included in future iterations of our ODE model.

Through our unbiased data-driven testing approach, we have integrated experimental data into a physiologically-relevant mathematical model exploring non-critical trabecular bone injury repair. A potential application of our modeling approach is to determine how bone healing times subsequent to injury can be improved via therapeutic intervention. Previous reports have demonstrated that modulating pro- and anti-inflammatory macrophages can alter the time taken for bone injury repair^14,19,50,89^. Because of the ability of the ODE model to recapitulate the temporal dynamics of the cellular populations involved in bone injury repair, we can investigate the precise timing at which to administer therapies in order to further shorten bone-healing time. Likewise, we can examine cellular behavior in response to a different sized injury, or even in a different bone injury context, such as non-union fractures. These points will be best addressed once we are able to enhance our ODE model with reciprocal mechanisms and fully couple the system.

In conclusion, we have developed an ordinary differential equation (ODE) model of osteoclast, osteoblast and bone dynamics, that considers the influences of polarized myeloid cells during trabecular bone injury. The model faithfully recapitulates bone volume dynamics during injury repair and returns to homeostasis. It further yields a number of novel insights regarding myeloid control of osteoclast- and osteoblast-mediated bone resorption and formation over time. To our knowledge, this model is the first to recapitulate longitudinal in vivo data of simultaneously measured bone and myeloid cell populations, as well as bone volume during bone healing. A better understanding of bone healing will have clinical translatability, allowing, for instance, accelerating the process and improve patient outcomes.

## Materials and methods

### Intratibial bone injury model

All animal studies were designed and performed in accordance with Guidelines for the Care and Use of Laboratory Animals published by the National Institutes of Health, and approved by the University of South Florida’s Institutional Animal Care and Use Committee under IACUC Protocol R5857-CCL. Additionally, studies abided by relevant ARRIVE guidelines. 5–6-week-old male immune-competent C57BL/6 mice were purchased from Jackson Laboratory with consideration for study statistical significance and power (n = 30). Surgically prepared mice (n = 25) were systematically sterilized with chlorhexidine. For each mouse, the knee joint was flexed to a 90° angle. Using a drilling movement, a 28G needled (0.3062 mm diameter) was inserted at a 0° angle, into the joint surface through the patellar tendon and tibial plateau in order to enter the intramedullary canal of the tibia. The depth of each injection was approximately 1 cm to ensure a substantial VOI for subsequent histological analyses. This needle action induced trabecular bone disruption and displacement without further compromising cortical bone structure beyond the needle insertion site (Fig. 1b). This method minimized bone-repair contributions from infiltrating extramedullary populations and allowed for interrogation of strictly bone marrow populations in subsequent analyses. Five uninjured mice were used as baseline controls. Mice receiving intratibial injuries were randomly selected and euthanized at days 1, 2, 3, 7 and 14 (n = 5/time point) for histological and flow cytometry analyses. Histological and FACS data were obtained in a blind manner to parameterize subsequent mathematical models.

### Micro-computed tomography

Injured tibias harvested from mice from all time points were centralized and were subjected to micro-computed topography (μCT) scanning using Scanco μ35 scanner to derive bone volume data. Individual bone scans were deidentified using numerical codes during, and reidentified following data analysis in a blinded fashion. A gap of 100 μm from the tip of growth plate towards the midshaft was avoided to ensure the high bone density nature of the growth plate does not mask potential differences in bone volume associated with the injury. Each bone was then scanned every 6 μm for a total span of 1000 μm along the midshaft (i.e. beginning approximately 16 slices away from the tip of the growth plate and analyzed for 180 cross-sections total/bone). Trabecular bone histomorphometry was subsequently performed after contouring each slice scan and reconstructing the 3-dimension volume of interest (VOI) structure of each bone using the built-in morph function (n = 30 bones; 5/time point). This process was performed repeatedly using different contours to generate bone status dynamics of the whole trabeculae (variable depending on bone), the region surrounding the injury (16 μm^2^ circle per contour), and of the injury itself (3μm^2^ circle per contour) (Supplemental Fig. 1). The positioning of contours across consecutive sections were each examined visually to ensure that the injury site was contained and centered within the VOI.

### TRAcP staining

Tibia bones from all time points were decalcified with 14% EDTA every other day for 3 weeks for further staining quantitation and analyses following μCT scans. Formalin fixed paraffin embedded (FFPE) bones were sectioned at 4 μm thickness to ensure single-cellular layer. Multiple slides sectioned at different depths from each bone were pooled for all time points, and were baked at 42 °C overnight to improve adhesion while retaining enzymatic activity for tartrate-resistant acid phosphatase (TRAcP) enzyme-based staining for osteoclast numbers. Deparaffined and rehydrated sections were pre-incubated in basic stock solution with napthol-ether substrate for 1 h at 37 °C and developed in pararosaniline dye and sodium nitrite for 10mins, also at 37 °C. Sections with red osteoclasts were further counterstained with hematoxylin to visualize bone tissue morphology. Fixed slides were imaged at 20X using Evos Auto brightfield microscopy to include injury site and its immediate periphery. All TRAcP positive (red) multinucleated osteoclasts within 5 μm radius from injury were counted, and mathematically converted to osteoclasts/bone marrow volume (#OCL/μm^3^) for each slide for each bone at each time point. This region of is consistent in area with the μCT analysis parameters to ensure consistency in data.

### Immunofluorescence staining and quantitation

Additional FPPE tibia bone sections were baked at 56 °C in preparation for immunofluorescence staining of osteoblast (RUNX2 at 1:500; Abcam Cat. No. ab81357), pro-inflammatory cells (NOS2 at 1:100; Abcam Cat. No. ab178945), anti-inflammatory cells (ARG1 at 1:200; Abcam Cat. No. ab133543) and nuclear staining (DAPI). Slides were processed in batch similar to TRAcP staining methodology. Deparaffined and rehydrated slides were subject to heat-induced antigen retrieval method. Sections were then blocked and incubated in primary antibodies diluted in 10% normal goat serum in TBS overnight at 4 °C. Subsequently, slides were stained with secondary Alexa Fluor 488 or 568-conjugated antibody at 1:1000 at room temperature for 1 h under light-proof conditions. Stained slides were stained with DAPI for nuclear contrast and mounted for imaging at 20X using Zeiss upright fluorescent microscope to include the injury site as well as the immediate peripheral tissue. All RUNX2 positive cells (red staining colocalizing with DAPI) within 5 μm radius from injury were counted and mathematically converted to osteoblasts / bone marrow volume (#OBL/μm^3^) for each bone at each time point. Again, this methodology ensured consistency across all acquired histological datasets.

### Flow cytometry and analysis

Harvested contralateral injured tibias (n = 30; 5/time point) had ends removed and were subjected to centrifugation at 16,000 g for 5 s for isolation of whole bone marrow for flow cytometry staining and analysis. Red blood cells were lysed using RBC Lysis Buffer from Sigma Aldrich (Cat. No. R7757-100ML) as per manufacturer’s guidelines. Live bone marrow cells were subject to FcR-receptor blocking (1:3; BioLegend; Cat. No. 101319) and viability staining (1:500; BioLegend; Cat. No. 423105). Samples were then stained by cell-surface conjugated antibodies from BioLegend diluted in autoMACS buffer (Miltenyi; Cat. No. 130-091-221) for phenotyping myeloid cells: CD11b-BV786 (1:200; Cat. No. 101243), LY-6C-Alexa Fluor 488 (1:500; Cat. No.128021) and LY-6G-Alexa Fluor 700 (1:200; Cat. No. 561236). Cells were then fixed with 2% paraformaldehyde in dark prior to intracellular staining. Fixed cells were permeabilized using intracellular conjugated antibodies to assess polarization status: NOS2-APC (1:100; eBioscience; Cat. No. 17-5920-80) and ARG1-PE (1:100; R&D; Cat. No. IC5868P). Appropriate compensation and fluorescence-minus-one (FMO) controls were generated in parallel either with aliquots of bone marrow cells or Rainbow Fluorescent Particle beads (BD Biosciences; Cat. No. 556291). All antibody concentrations were titrated prior to injury study using primary bone marrow cells to ensure optimal separation and detection of true negative and positive populations. Stained controls and samples were analyzed using BD Biosciences LSR flow cytometer (Supplemental Fig. 4). All datasets were batch analyzed to ensure optimal consistent gating stringency.

## Mathematical and computational methods

### Model parameterization

*Initial ODE model* The initial Osteoblast/Osteoclast/Bone ODE model is presented in equation Fig. 2a. OB, OC and B represent osteoblasts, osteoclasts and bone volume, respectively. Osteoblast and osteoclast equations are composed of a homeostatic source term, a clearance term, and an injury-triggered expansion term. The osteoblast clearance parameter δ_OB_ was fixed from literature and in order to ensure osteoblast homeostasis level, the source term H_OB_ was fixed at δ_OB_*OB_0_, where OB_0_ represents the initial level of osteoblasts. The osteoblast proliferation rate γ_OB_ and duration of expansion T_anab_ were calibrated in fitting the osteoblast dynamics to the experimental data. The osteoclast decrease rate (Inhib_OC_), the decrease duration (T_antiCatab_), the replenishment time (T_Catab_) and the replenishment rate (R_OC_) were calibrated in fitting the osteoclast dynamics to the experimental data. The homeostatic clearance parameter was fixed to R_OC_/OC_0_ in order to ensure homeostasis back to the initial osteoclast level.

The bone equation comprises two terms: a bone resorption term, proportional to the number of osteoclasts and proportional to bone volume, and a bone formation term, proportional to osteoblast number. The resorption term is proportional to bone volume since less bone translates to less bone available for osteoclast resorption. On the other hand, osteoblast-mediated bone formation is independent of available bone. A range of possible resorption rates was derived from published measurements. Equation on Fig. 2a **#** shows how bone formation parameter is fixed at δ_B_OC_0_B_0_/OB_0_, where B_0_ is the initial bone level, in order to ensure that bone level remains at homeostasis when osteoclast and osteoblast levels are at homeostasis (Corresponding predictions on Fig. 2c). Equation on Fig. 2a **&** shows the case where both δ_B_ and Π_B_ are freely optimized (corresponding fit on Fig. 2d).

*Piecewise linear temporal variation of bone resorption and formation rates* The initial ODE model was further used to study time-dependent bone resorption rate. It was defined as an explicit piecewise linear function of time (Supplemental Figs. 7a and 8a). The dynamics of osteoclast and osteoblast was imposed from the previous fit. The successive slopes of the piecewise linear function and the initial resorption rate were all estimated from fitting the resulting bone dynamics to bone experimental data. The parameter space for the optimization was defined as follows: The slopes were allowed to be positive or negative, with the constrain that the resorption rate cannot become negative or go beyond the upper bound defined from literature. Using the same approach, model sought to recapitulate bone dynamics by optimizing osteoblast activity dynamics (Supplemental Fig. 8).

*Enhanced ODE model including polarized monocytes/macrophages* For the polarized macrophage part (Fig. 4), cells clearances/lifespans were fixed from literature and all the other parameters were calibrated to fit the experimental data. The osteoblast and osteoclast fits were kept the same as in the initial model. For the bone equation, homeostatic bone resorption rate δ_B_ (before and after injury), pro-inflammatory monocytes/macrophages-mediated bone resorption stimulation parameter (α) and anti-inflammatory macrophages-mediated bone formation stimulation parameter (β) were all calibrated to fit the experimental bone dynamics. The homeostatic bone formation rate Π_B_ was fixed such that Π_B_ = δ_B_OC_0_B_0_/OB_0_ so bone level is ensured to remain at homeostasis when osteoclast and osteoblast levels are at homeostasis in absence of injury (no polarized monocytes and macrophages).

### ODE solver

The ODE45 function of Matlab was used to solve the differential equation system. The experimental baseline values (time 0) were used as initial conditions.

### Parameter estimation method

To estimate parameters facilitating goodness of fit, we defined the following objective function:

$$LS\left( \alpha \right) = Max_{{\left\{ j \right\}}} \mathop \sum \limits_{i = 1}^{N} \frac{{\left( {f_{j} \left( {t_{i} ,\alpha } \right) - D_{ij} } \right)^{2} }}{{\sigma_{i}^{2} }}$$

where i represents the time point index and j the variable index, α represents the parameter set used to evaluate the model function f, $D_{ij}$ represents the experimental data of variable j at time point i, and $\sigma_{i}$ represents the experimental error. The choice of this functional form instead of the sum of the squares of the residuals was motivated to avoid that one fit variable would be “sacrificed” to the benefit of another one. This way, we ensure that all variables are equally well fitted.

- In order to minimize this function representing the error estimate between data and model, we used the Matlab function fminsearch with a penalization term to stay in a parameter range set with reasonable boundaries.
- AIC criterion is defined as follows:
  $$AIC\left( \alpha \right) = 2p + LS\left( \alpha \right)$$
  where p is the number of parameters.

## Supplementary Information

Supplementary Information.
